# Analysis of State-Level Drug Pricing Transparency Laws in the United States

**DOI:** 10.1001/jamanetworkopen.2019.12104

**Published:** 2019-09-25

**Authors:** Martha S. Ryan, Neeraj Sood

**Affiliations:** 1 Sol Price School of Public Policy, University of Southern California, Los Angeles; 2 Schaeffer Center for Health Policy & Economics, University of Southern California, Los Angeles

## Abstract

This cross-sectional study examines state drug pricing transparency laws enacted to increase supply chain price transparency in the United States.

## Introduction

Increasing drug prices strain the budgets of consumers and public payers, including Medicaid. Faced with an opaque drug supply chain, few people outside of the pharmaceutical industry know which supply chain participants are making excess profits and where to target policy solutions.^1,2^ Some states have responded by passing laws to increase drug price transparency in the supply chain.^3^ In this study, we analyzed state drug price transparency laws to assess their potential to improve transparency in the drug supply system. Prior research has focused on state laws requiring manufacturers to report research and development expenditures and efforts to control costs through drug importation or manufacturer price controls.^3,4^ To our knowledge, this is the first study to focus on state laws to improve drug price transparency across the supply chain.

## Methods

In this cross-sectional study, we searched the National Conference of State Legislatures prescription drug database^5^ for transparency laws, restricting our search to laws enacted between January 1, 2015, and December 31, 2018. We coded each law by which entity in the drug distribution system (ie, manufacturers, wholesalers, pharmacy benefit managers [PBMs], pharmacies, insurers, or a combination) was required to provide the information. We also evaluated whether laws would reveal previously unavailable information in the form of profits or real transaction prices, including rebates and concessions, for supply chain participants. Because knowing real transaction prices is crucial to determining who is earning excess profits in the supply chain and for targeting policy solutions, we labeled transparency laws resulting in new disclosure of this information as informative; laws mandating reporting of information that was already available from other sources, including wholesale acquisition costs, list prices, or other intermediary prices, were labeled uninformative.^2^ The methods for how we labeled each law are shown in the eTable in the Supplement. We followed the Strengthening the Reporting of Observational Studies in Epidemiology (STROBE) reporting guideline for cross-sectional studies. Institutional review board approval was not required because the study used only public data on state laws and not human participants as defined by Common Rule 45 CFR part 46.102. Data analysis was conducted from October 1, 2018, to February 2, 2019.

## Results

Of 166 drug pricing laws identified, 35 laws passed in 22 states included a transparency component, but only 7 laws passed in 6 states were deemed informative (Table 1). Two states required that net price be reported—by insurers in Vermont and by manufacturers in Maine. Only Oregon and Nevada passed laws that required that profits be reported by manufacturers. Laws in Connecticut, Louisiana, and Nevada required that PBMs report rebates in aggregate but not at the individual-drug level. Uninformative transparency laws commonly required that supply chain participants disclose pricing or formulary design methodology, provide advance notice of list price increases, or register with a government regulatory body.

**Table 1.** Reporting Requirement Content in Transparency Laws

Characteristic	States, No.
Informative	6
Requirement	
Manufacturers must report net price or profits	3^a^
Wholesaler must report net price or profits	0
Pharmacy must report net price or profits	0
Pharmacy benefit manager must report rebates or profits	3^b^
Insurer must report net price or profits	1^c^

^a^ Includes Maine S 484, Nevada SB 539, and Oregon 4005.

^b^ Includes Connecticut H 5384, Louisiana SB 283/282, and Nevada SB 539.

^c^ Includes Vermont S 92.

Most states with transparency laws targeted PBMs (15 states) or insurers (11 states); 2 states passed laws that targeted pharmacies, and no states passed laws that targeted wholesalers (Table 2). Only Nevada and Vermont passed laws that targeted 3 distinct supply chain segments (ie, manufacturer, insurer, and PBM); no state passed laws that targeted more than 3 supply chain segments. Importantly, no state passed laws that together revealed true transaction prices or profits across all supply chain segments.

**Table 2.** Number of States Targeting Different Types of Entities Through Transparency Legislation

Target of Reporting Requirement	States, No.^a^
Pharmacy benefit managers	15
Insurers	11
Manufacturers	8
Pharmacies	2
Wholesalers	0

^a^ Total number of states exceeds 35 because several states passed multiple laws, and state reporting requirements may target multiple segments of the supply chain.

## Discussion

The process of manufacturing, distributing, and paying for pharmaceuticals involves numerous commercial entities, including drug manufacturers, wholesalers, pharmacies, PBMs, and insurers.^6^ Real transaction prices, including rebates and discounts at each stage of this process, are needed to understand profits and distribution of rents across these supply chain segments. Without this information, regulators cannot target policy solutions at the segments making excessive profits.

Despite many recent state laws about price transparency, we found that most of them were insufficient to reveal true transaction prices, and no state passed legislation that provided effective transparency across the entire supply chain. To ensure drug price legislation is useful, policy makers should require that real price information, including discounts and rebates, is reported by all supply chain participants. Ideally, this information should be available for drugs with the largest effect on the state’s budget or largest price increases. If requiring such disclosure at the individual-drug level would invite legal challenge, states should at least require each supply chain segment to report aggregate profits from sales in that state.

## Notes

Supplement. **eTable.** Method of Classifying Laws **eReferences.**
