# Errors in the Supplement

**DOI:** 10.1001/jamanetworkopen.2019.15280

**Published:** 2019-10-23

In the Research Letter titled “Analysis of State-Level Drug Pricing Transparency Laws in the United States,”^1^ published September 25, 2019, in the first column of the eTable in the Supplement, the first row should have been labeled “State & Bill/Rule/Statute”; row 12, “KY SB 117”; row 22, “MD 15-1628.1”; row 24, “MN 151.214”; and row 36, “VT S 92/A 193.” In the second column, row 23 should have read “2018.” This article has been corrected.^1^
