# Supplementary material for: Analysis of State-Level Drug Pricing Transparency Laws in the United States
Source: JAMA Netw Open. 2019 Sep 25;2(9):e1912104. doi: 10.1001/jamanetworkopen.2019.12104 (PMC6763978; doi:10.1001/jamanetworkopen.2019.12104)
Supplement: Supplement. — eTable. Method of Classifying Laws eReferences. [file jamanetwopen-2-e1912104-s001.pdf]

Supplementary Online Content

Ryan MS, Sood N. Analysis of state-level drug pricing transparency laws in the United States. *JAMA Netw Open*. 2019;2(9):e1912104.
doi:10.1001/jamanetworkopen.2019.12104

eTable. Method of Classifying Laws
eReferences.

This supplementary material has been provided by the authors to give readers additional information about their work.

© 2019 Ryan MS et al. *JAMA Network Open.*

eTable. Method of Classifying Laws

State & Bill	Year	Target(s)	Net price	Rebates	Profit Margin				Informative?[a]
					Whole-saler	PBM	Manu-facturer	Pharmacy	
AK HB 240	2018	PBMs	No	No	No	No	No	No	No
AR S 466	2015	Insurers	No	No	No	No	No	No	No
AR A 815	2017	Insurers	No	No	No	No	No	No	No
CA SB 17	2017	Manufacturer, Insurer	No	No	No	No	No	No	No
CT H 5384	2018	Manufacturers, PBMs, Insurers	No	Yes	No	No	No	No	Yes
CT S 811	2015	Insurers	No	No	No	No	No	No	No
DE HB 284	2016	PBMs	No	No	No	No	No	No	No
FL HB 589	2017	Pharmacies	No	No	No	No	No	No	No
HI H 252	2015	PBMs	No	No	No	No	No	No	No
HI H 261	2015	Insurers	No	No	No	No	No	No	No
KY KRS 304.17A-162	2016	PBMs	No	No	No	No	No	No	No
KY SB 5	2018	PBMs	No	No	No	No	No	No	No
LA H 436	2017	Manufacturers	No	No	No	No	No	No	No
LA SB 59	2017	PBMs	No	No	No	No	No	No	No
LA H 568	2015	Pharmacies	No	No	No	No	No	No	No
LA SB 282	2018	Insurers	No	Yes	No	No	No	No	Yes
LA SB 283	2018	PBMs	No	Yes	No	No	No	No	Yes
ME S 484/ LD 1406	2018	Manufacturers	Yes	No	No	No	No	No	Yes
ME S 229	2015	Insurers	No	No	No	No	No	No	No
ME H 788	2016	PBMs	No	No	No	No	No	No	No
MD Article 15-1628.1	2015	PBMs	No	No	No	No	No	No	No
MD 15-1628.1	2017	PBMs	No	No	No	No	No	No	No
MN Statute 151.214	2017	PBMs	No	No	No	No	No	No	No
MT S 211	2015	PBMs	No	No	No	No	No	No	No
NV SB 539	2017	Manufacturers, PBMs, Insurers	Yes	Yes	No	No	Yes	No	Yes
NH HB 1418	2018	Manufacturers	No	No	No	No	No	No	No
NJ N.J.A.C. 11:4-62	2017	PBMs	No	No	No	No	No	No	No
OR H 4005	2018	Manufacturer, Insurer	No	No	No	No	Yes	No	Yes
PA A 169	2016	PBMs	No	No	No	No	No	No	No
RI S 2467	2016	PBMs	No	No	No	No	No	No	No
SD S 118	2015	Insurers	No	No	No	No	No	No	No
TX H 1624	2015	Insurers	No	No	No	No	No	No	No
TX H 1227	2017	Insurers	No	No	No	No	No	No	No

© 2019 Ryan MS et al. *JAMA Network Open.*

VT S 216 / A 165	2016	Manufacturer, Insurer	No	No	No	No	No	No	No
VT S 92 / Act 193	2018	Manufacturers, Insurers, PBMs	Yes	No	No	No	No	No	Yes

[a] We classify each law as informative if it requires a supply chain participant (such as insurers, PBMs, manufacturers) to disclose either 1) rebate amount, 2) profit, or 3) net price. We base these criteria on research findings that rebates, profits and net prices are crucial to determining who is responsible for high drug costs and for examining the potential effects of policy proposals to control drug costs, including removing safe harbor protections for rebates, using external reference pricing and eliminating spread pricing. [1,2,3,4,5] To corroborate our approach, we asked a second analyst unfamiliar with our study to independently re-code our sample of laws using the same criteria. This second coding agreed with our original coding in 35 out of 35 cases.

eReferences.

1. Wineinger NE, Zhang Y, Topol EJ. Trends in Prices of Popular Brand-Name Prescription Drugs in the United States. *JAMA Network Open.* 2019;2(5):e194791-e194791.
2. Sood N, Shih T, Van Nuys K, Goldman DP. Follow The Money: The Flow Of Funds In The Pharmaceutical Distribution System. *Health Affairs Blog.* 2017. https://www.healthaffairs.org/do/10.1377/hblog20170613.060557/full/. Accessed June 13, 2019.
3. Dusetzina SB, Conti RM, Yu NL, Bach PB. Association of Prescription Drug Price Rebates in Medicare Part D With Patient Out-of-Pocket and Federal Spending. *JAMA Internal Medicine.* 2017;177(8):1185-1188.
4. Council of Economic Advisers, Executive Office of the President. Reforming Biopharmaceutical Pricing at Home and Abroad. https://www.whitehouse.gov/wp-content/uploads/2017/11/CEA-Rx-White-Paper-Final2.pdf. Published February 9, 2018. Accessed July 1, 2019.
5. Senate Committee on Finance. Description of the Chairman’s Mark: The Prescription Drug Pricing Reduction Act (PDPRA) of 2019. https://www.finance.senate.gov/imo/media/doc/FINAL%20Description%20of%20the%20Chairman's%20Mark%20for%20the%20Prescription%20Drug%20Pricing%20Reduction%20Act%20of%202019.pdf. Published July 25, 2019. Accessed July 27, 2019.

© 2019 Ryan MS et al. *JAMA Network Open.*
